# Can conceptual beliefs shape impressions from voices?

**DOI:** 10.3758/s13423-026-02876-w

**Published:** 2026-03-31

**Authors:** Mairi Irvine, Nadine Lavan

**Affiliations:** 1 Department of Biological and Experimental Psychology, School of Biological and Behavioural Sciences, Queen Mary University of London, London, UK; 2 Centre for Brain and Behaviour, School of Behavioural and Biological Sciences, Queen Mary University of London, Mile End Road, London, E1 4NS UK

**Keywords:** Conceptual beliefs, First impressions, Voices, Stereotypes, Representational similarity analysis, Top-down

## Abstract

When we hear a voice, we routinely form an impression of the speaker’s personality and characteristics. Previous work has primarily modelled first impression formation as a bottom-up, feed-forward process, where acoustic cues drive how different person characteristics, such as trustworthiness or competence, are perceived from unfamiliar voices. Contributions from top-down processes to impression formation, such as the influences of social stereotypes and other conceptual beliefs, have to date received less attention in voice research. Here, we quantify how much such top-down processes could contribute to first impression formation by mapping representational spaces for (1) first impression judgments from voices and (2) conceptual beliefs about person characteristics in other people in a behavioural study. Using Representational Similarity Analysis (RSA), we observe substantial structural similarities between these spaces. This association shows that conceptual beliefs could play a role in shaping impressions, via top-down influences. This effect generalises across voice types (e.g., typical, AI-generated, pathological voices), listener samples, and even across modalities to facial impressions. Aligning with recent theoretical advances in social psychology, our findings thus call for vocal impression research to start to explicitly model and explore the interplay between top-down and bottom-up processes during impression formation.

**Supplementary Information:**

The online version contains supplementary material available at 10.3758/s13423-026-02876-w.

## Introduction

Humans routinely and efficiently form first impressions from voices: Hearing an unfamiliar voice for only a few milliseconds of hearing an unfamiliar voice is enough for listeners to make judgments about person characteristics, such as trustworthiness, dominance, competence, and attractiveness (Lavan, 2023b; McAleer et al., 2014; Mileva, 2025; Mileva & Lavan, 2023; Sutherland & Young, 2022). Impressions are to some degree shared among individuals, such that people, on average, agree whether a voice, for example, sounds trustworthy or not. At the same time, there are also idiosyncratic aspects of impressions, showing that personal preferences also shape how we evaluate others (Hehman et al., 2017; Lavan & Sutherland, 2024; Martinez et al., 2020). Even though first impressions are formed without having any existing knowledge about the other person and are thus often not accurate reflections of their true characteristics (Foo et al., 2022; Skuk et al., 2024; Todorov et al., 2015), they still influence our behaviours, decisions, and social interactions. As such, first impressions can, for example, predict election outcomes (Klofstad, 2016; Mileva et al., 2020; Schild et al., 2022; Tigue et al., 2012) and decisions of landlords about which tenants they choose to rent accommodation to (Purnell et al., 1999).

How do listeners form these first impressions and what shapes them? Theoretical models (Belin et al., 2004, 2011; Lavan & McGettigan, 2023) suggest that voices are processed along functional pathways, starting with low-level acoustic analysis, followed by a voice structural analysis, which determines whether a sound is a voice. Voices are then processed along pathways for speech, emotion, and identity perception, with the identity pathway presumably processing any kind of person-related information (Lavan & McGettigan, 2023). While models acknowledge bidirectional influences, allowing for bottom-up and top-down modulation across all stages of processing, these models have tended to be primarily described and tested in terms of bottom-up and feed-forward processes. As such, perceptual studies usually frame voices as a signal that carries (acoustic) information about a certain speaker characteristic that listeners then use to form an impression (be it accurate or not; Lavan, 2023b; McAleer et al., 2014; Mileva & Lavan, 2023; Sorokowski et al., 2023, 2024). Studies then, for example, test the bottom-up nature of impression formation by identifying which acoustic properties can predict first impressions from voices (Aung & Puts, 2020; Belin et al., 2017; Borkowska & Pawlowski, 2011; McAleer et al., 2014; Pisanski & Rendall, 2011; Ponsot et al., 2018; Puts et al., 2007). These studies have often yielded influential findings characterising how these bottom-up, feed-forward influences shape impressions. However, the relative preponderance of studies adopting this bottom-up processing framework has meant that the role of top-down influences (and any interaction between top-down and bottom-up influences) on impression formation has received relatively less attention in both empirical and theoretical voice research.

In social psychology, prior work examining how people infer traits or characteristics of others stresses that stereotypes and conceptual beliefs can influence (amodal) trait attributions via top-down processes (Dotsch et al., 2011; Newman & Uleman, 1990; Wigboldus et al., 2003). A theoretical framework, dynamic interactive theory (Freeman & Ambady, 2011; Freeman et al., 2020; Stolier, Hehman, Freeman et al., 2018a), builds on prior social psychology research. This model highlights the importance of both bottom-up and top-down processes during impression formation from faces, bodies, or voices. The model therefore predicts that first impressions do not only arise through perceiving and evaluating the acoustic features of a voice. Instead, impressions can also be shaped by stimulus-independent conceptual beliefs that are learned over time through, for example, repeated exposure to people in general, but also biased cultural narratives and media representations (e.g., Fiske & Taylor, 2016). Bottom-up and top-down processes are then thought to interact to give rise to the impressions we form. Studies of face perception find that first impressions formed from viewing faces are structured similarly to a purely conceptual trait space, mapping how conceptual beliefs about person characteristics relate to one another (e.g., the belief that trustworthy people are also warm; Oh et al., 2021; Stolier, Hehman, Keller et al., 2018b). Similarly, research has shown that contextual cues, manipulated by providing participants with additional information about an otherwise unfamiliar person (e.g., “this person served a prison sentence”), influence first impressions from faces (Coutts et al., 2024). Taken together, these findings, therefore, suggest that impressions are not only determined by information encoded in face stimuli but also share similarities with abstract conceptual beliefs that may have no direct link to the person being perceived, alongside other (contextual) information available to perceivers. While causality is difficult to unambiguously establish in these studies, these results suggest that impressions from faces are shaped by conceptual beliefs (alongside bottom-up, stimulus-driven processes).

While top-down influences on impression formation received only limited attention in voice perception, these kinds of influences are often, to some degree, acknowledged in studies by mentioning (but never thoroughly examining) that first impressions are likely shaped by stereotypes and social biases. Moreover, given the widely accepted parallels between face and voice perception, it is reasonable to assume that conceptual beliefs and other top-down processes similarly affect how we form impressions from voices. As such, the notion of top-down influences being important for first impressions formation has noted in empirical and theoretical voice research but has never been directly and systematically quantified.

The current study aimed to examine whether and how conceptual beliefs about other people could shape first impressions from voices. Through this, we work towards a more comprehensive understanding of how we evaluate others based on their voices, considering both bottom-up influences that are driven by stimulus properties and top-down influences such as conceptual beliefs potentially shaping impressions. We asked participants to complete two types of tasks: They first perceptually evaluated unfamiliar voices and faces for a set of person characteristics that are relevant to forming first impressions (*age, attractiveness, competence, dominance, educatedness, friendliness, health, masculinity, professionalism, and trustworthiness*; Lavan, 2023a; Lin et al., 2021; McAleer et al., 2014; Mileva, 2025; Oosterhof & Todorov, 2008; Stolier, Hehman, Freeman et al., 2018a, Stolier, Hehman, Keller et al., 2018b; Sutherland et al., 2013). Participants then completed a task in which they rated how likely all possible pairs of these 10 person characteristics are likely to co-occur in other participants (e.g., *“If a person is attractive, how likely are they to be healthy?”),* thus mapping participants’ conceptual beliefs about how person characteristics relate to one another. Using Representational Similarity Analysis (RSA), we were able to chart out the structure of these multivariate and intercorrelated perceptual and conceptual impression spaces and relate the two spaces to one another. Considering findings from face impressions, we predicted that the structure of conceptual beliefs would share similarities with the structure of voice impressions.

As a secondary research question, we probed the generalisability and possible constraints of whether and how much conceptual beliefs and first impressions formed from voices share information. To do this, we replicated and ran the same tasks we had run for “typical” voices, using 1) images of faces and 2) diverse voice types (specifically, AI-generated and pathological voices). This enabled us to directly assess the generalisability of our findings across modalities (typical voices vs typical faces) and different kinds of voices (typical vs diverse voices). Stronger relationships for any specific modality or stimulus type would indicate more scope for conceptual beliefs to shape first impressions within a specific set of stimuli, while weaker relationships would have indicated the opposite.

## Methods

### Participants

A total of 369 participants (*M*_age_ = 40.39 years, *SD* = 12.06, 183 women) were included in the analysis for this study. All participants were recruited from the online participant recruitment service, Prolific.com, were native speakers of English, based in the US, had no self-reported hearing difficulties (for all experiments involving voice stimuli) and had normal or corrected-to-normal vision (for the experiment involving face stimuli). The experiment was approved by the local ethics board (PSY2024-69). Participants were reimbursed for their time at a rate of at least £9 per hour.

Of the 369 participants, 95 participants (*M*_age_ = 39.78 years, *SD* = 11.86, 53 women) evaluated typical voices, 92 participants (*M*_age_ = 40.94 years, *SD* = 12.45, 48 women) evaluated typical faces, 90 participants (*M*_age_ = 40.75 years, *SD* = 11.67, 43 women) evaluated AI-generated voices, and 92 participants (*M*_age_ = 40.85 years, *SD* = 12.72, 39 women) evaluated pathological voices. This resulted in 23–31 ratings per stimulus (mean = 27.35 ratings, *SD* = 1.94; mode = 27).

Before arriving at this final sample, data from some participants were excluded: Two participants failed more than 20% of attention checks, such that all data from these participants were excluded (see Procedure). Twenty-six participants gave the same response on over 80% of trials in one or more tasks, such that we excluded data from the affected tasks only (one task for 23 participants, two tasks for two participants, three tasks for one participant), but their remaining data were retained for group-level RSA. For individual-level analyses, we additionally excluded all data from participants who showed this response pattern in conceptual belief ratings (*N* = 17) or for perceptual ratings of more than one person characteristic (*N* = 1), as these data were essential to compute our analysis.

### Stimuli

#### Voices

We included three types of voices in our study: “Typical” adult voices, AI-generated voices, and pathological voices. For the perceptual ratings of “typical voices,” 80 voices (20 men, 60 women; aged 19–63 years, *M*_age_ = 31 years, none labelled as having a voice pathology) were selected from the Perceptual Voice Qualities Database (Walden, 2022). All speakers had Northern American accents and produced short sentences with neutral linguistic content (e.g., “My mom makes lemon muffins”; “We eat eggs every Easter.”). We included more female voices in our study as a result of practical constraints: The Perceptual Voice Qualities Database included only 21 typical male voices, one of which had to be excluded due to recording quality issues, while many more female voices were available. To ensure that the different experimental conditions (outlined below) were as comparable as possible, we constrained the number of male voices and faces to 20 across all conditions.

For the perceptual ratings of “AI-generated voices,” we generated 80 voices (20 male, 60 female) using the VoiceDesign v3 feature from ElevenLabs. In VoiceDesign v3, users can generate and design voices for text-to-speech synthesis based on written prompts (example prompt from the ElevenLabs website: “The friendly mythical God, Zeus, with a huge, deep, powerful voice. Charming, proud, strong, and theatrical.”). To match the sample of AI-generated voices to the typical voices above in terms of their demographic make-up, we used the reported gender, age in years, and accent of each voice included in the typical sample as text prompts (e.g., “Male voices, 32 years old, North American accent”) to generate recordings from 80 AI-generated voices producing the same sentences as the typical voices.

For the perceptual ratings of “pathological voices,” 80 voices (29 male, 51 female; aged 14–90 years, *M*_age_ = 55 years) were selected from the Perceptual Voice Qualities Database (Walden, 2022). The pathological voices were therefore substantially older on average than the other two voice samples. All selected voices had a readily perceptible voice pathology (mean CAPE-V severity score = 60.49; *SD* =18.3; range: 30–99), such as vocal fold paralysis, lesions, tremors, or polyps. The severity of pathology included thus ranged from mild-to-moderate to severe (Walden, 2025).

All recordings were trimmed to the start and end of the spoken sentences, were root-mean-square (RMS) normalised for intensity using PRAAT (Boersma & Weenink, 2023), and had a duration of 1–4 s.

#### Faces

The stimuli for the perceptual ratings of “typical faces” were selected from the 10k US Adult Faces Database (Bainbridge et al., 2013), a well-used database of naturalistic face images taken from the internet. We selected 80 images (20 male, 60 female). Faces are shown with an oval mask and on a white background. All images showed that faces that were looking at the camera, with a neutral expression, were front-facing, and the images were rated to be of good quality. No constraint on the ethnicity, age, or any other characteristics of the face images according to the background measures provided for the database used.

### Procedure

The experiment was run using Gorilla Experiment Builder (Anwyl-Irvine et al., 2020). Before completing the first task, participants read an information sheet and then provided informed consent.

Participants then completed a perceptual ratings task in which they rated sets of faces or voices for 10 person characteristics (masculinity, age, health, attractiveness, dominance, competence, friendliness, trustworthiness, professionalism, and educatedness*).* There were four versions of the main experiment, which differed only in terms of which stimuli (typical voices, typical faces, AI-generated voices, and pathological voices) and associated instructions in the perceptual ratings task. This was followed by a task which measured the conceptual beliefs about how these different person characteristics relate to one another.

The 10 person characteristics have been frequently used in first impression research from voices and faces, include a range of apparent (masculinity, age, health) and largely inferred (trustworthiness, competence) characteristics (Lavan, 2023a, b; Lavan et al., 2024; Lavan & Sutherland, 2024; Lin et al., 2021; McAleer et al., 2014; Mileva, 2025; Stolier, Hehman, Freeman et al., 2018a, Stolier, Hehman, Keller et al., 2018b; Sutherland et al., 2013). We used the same 10 characteristics for all voice and face impressions to ensure that our analyses and findings were comparable across different stimulus types. We were careful to choose characteristics that were readily applicable to all characteristics (e.g., avoiding modality-specific characteristics, such as “well-spoken” or “baby-faced”). It is, of course, possible that these 10 characteristics may be differentially salient or important for first impression from the different stimuli used (e.g., “health” will likely be more salient as a percept for pathological voices than for the other stimulus types). We, however, reasoned that these differences in salience should mainly affect mean ratings (e.g., overall lower ratings of “health” for pathological voices; see Supplementary Fig. 1). Below, we will outline the details for these first perceptual ratings tasks.

### Perceptual impressions of person characteristics

Participants first completed the perceptual ratings task in which they were presented with either the set of 1) typical voices, 2) typical faces, 3) AI-generated voices, or 4) pathological voices were asked to rate these stimuli for three of the 10 person characteristics included in this study on a 9-point Likert scale (1 = *not at all [characteristic]*, 9 = *very [characteristic]*, with 1 denoting “sounds very feminine” and “sounds like a young adult” for the masculinity and age scales, respectively). For the versions of the experiment including AI-generated voices and pathological voices, participants were additionally given information about the nature of the voices they would hear (“You will hear short recordings of *AI-generated voices*. This means that these are not voices of real people but voices that were generated by a computer”; “You will hear short recordings of *pathological voices*. This means that the people have issues that affect their voice, such that it may sound hoarse, thin, shaky, or otherwise different from ‘normal.’”).

The ratings for the three person characteristics were presented in a blocked design, such that a participant might first have rated all 80 stimuli for attractiveness, followed by dominance, followed by educatedness. The task was self-timed, the stimuli within each block and the order of the three blocks were presented in fully randomised order. We created 10 triplets of characteristics, one of which participants were randomly assigned and which determined which of the 10 person characteristics each participant rated.

Each of the three ratings blocks included eight vigilance trials (10% of experimental trials) in which participants were either presented with a voice recording (for all voice ratings) or text written (for face ratings) on the screen that instructed them to provide a specific rating (“Please select 7”). The vigilance trials were randomly interspersed between the experimental trials. These vigilance trials served two purposes:

1. Ensuring that participants paid attention throughout the experimental tasks, and
2. Functioning as checks to confirm that participants were able to hear the voices and see the faces appropriately, since participants can only give a correct rating if they were able to hear or see the vigilance stimulus.

#### Conceptual beliefs task

In the conceptual beliefs task, participants were shown written questions on the screen relating to a participant’s beliefs about how likely two of the ten person characteristics would co-occur in other people (e.g., *“If a person is attractive, how likely are they to be healthy?”*). Participants responded to this question again via a 9-point scale ranging from “not likely at all” to “very likely.” There were 90 trials in this task to cover all possible pairwise permutations of the 10 person characteristics, such that each of the 45 possible pairs was presented in two orders (e.g., “healthy”–“attractive” and “attractive”–“healthy). The order of trials was fully randomised, and the task was self-timed.

As for the perceptual ratings task, nine vigilance trials for which participants were presented with text written on the screen that instructed them to provide a specific rating for each vigilance trial.

#### Group-level representational similarity analysis (RSA)

To quantify how much conceptual beliefs contribute to the formation of first impressions based on voices (and faces) at a group level, we ran a representational similarity analysis (RSA). RSA is a powerful methodological tool which allows for the direct comparison of different multivariate spaces (e.g., conceptual beliefs and perceptual impressions here) across different modalities and stimulus types (Kriegeskorte & Kievit, 2013; Kriegeskorte et al., 2008; Popal et al., 2019).

**Fig. 1:**
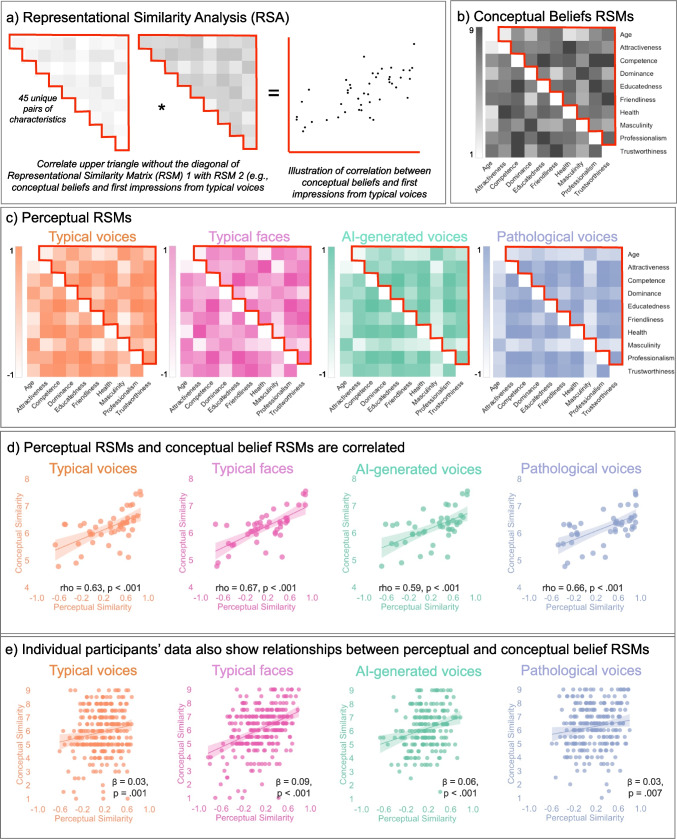
Relationships between perceptual impressions and conceptual beliefs. **a)** Illustration of the RSA approach, where we correlate the upper triangles of two RSMs to establish **b)** Conceptual belief RSM: Heatmap showing conceptual similarity across the pairs of person characteristics used in this study (e.g., age, attractiveness, and competence; 1 = not likely to co-occur, 9 = very likely to co-occur). **c)** Perceptual RSMs: Heatmaps showing similarity of perceptual impressions formed from typical voices, typical faces, AI-generated voices, and pathological voices (Spearman’s rho, −1 = two person characteristics are negatively correlated, 1 = two person characteristics are positively correlated. **d)** Group-level RSA correlations: Scatterplots showing significant correlations between upper-triangles of the conceptual RSMs and the perceptual RSMs for all four stimulus types included in this study (rho range = 0.59–0.67, all *p* <.001), shading around the trendlines are 95% confidence intervals. **e)** Individual-level correlations: Scatterplots showing significant relationships between perceptual and conceptual RSMs at a participant-level (β range = 0.03–0.09, all *p* ≤.007). See Supplementary Fig. 2 for additional visualisations of the relationships from the RSMs in panel b) and c). (Colour figure online)

We created four 10 × 10 perceptual representational similarity matrices (RSMs) based on perceptual impressions for 1) typical voices, 2) typical faces, 3) AI-generated voices, and 4) pathological voices by computing average ratings per stimulus for each person characteristic and correlating these item-wise average ratings exhaustively across person characteristics, using Spearman correlations (Fig. 1c). We use Spearman correlations throughout, as some of our data were not normally distributed (e.g., masculinity ratings across all stimuli, see Supplementary Fig. 1). We then created one 10x10 conceptual RSM based on the conceptual beliefs ratings, by averaging across the two trials, including the same pair of characteristics (e.g., “healthy”–“attractive” and “attractive”–“healthy) for each participant and then averaging again across participants to compute a mean rating for each pair of characteristics. These mean ratings were also entered into the 10 × 10 RSM mapping conceptual beliefs (Fig. 1b). These perceptual and conceptual RSMs were both symmetrical around the diagonal and can describe the representational structure for how different person characteristics relate to one another. Since first impressions are inherently multivariate, where individual characteristics (e.g., age, attractiveness) are intercorrelated (Lin et al., 2021; McAleer et al., 2014; Mileva, 2025; Oosterhof & Todorov, 2008; Sutherland et al., 2013), RSA is an ideal method to query impressions as it enables researchers to map how different variables (here, perceptions of the 10 characteristics) relate to one another without having to selectively investigate individual characteristics.

To answer our research questions, we then vectorised the upper triangles of two of the RSMs (thus omitting redundant values as well as the diagonal) and ran Spearman’s correlations of the two resulting vectors of perceptual and conceptual similarity values for the 45 unique pairs of characteristics included in our study (Fig. 1a). Specifically, to assess whether and to what degree conceptual beliefs shape impressions from typical voices, we correlated the RSM for impressions from typical voices and the RSM for conceptual beliefs. We then replicated the analysis from Stolier, Hehman, Freeman et al. (2018a), Stolier, Hehman, Keller et al. (2018b), assessing whether and to what degree conceptual beliefs shape impressions from typical faces by correlating the RSM for impressions from typical faces and the RSM for conceptual beliefs. We next asked whether and to what degree conceptual beliefs also shape impressions from other types of voices by correlating the perceptual RSMs for impressions from AI-generated and pathological voices, respectively, and the RSM for conceptual beliefs.

#### Individual-level analysis

Each participant completed the conceptual beliefs task, alongside perceptual ratings for three person characteristics (e.g., age, trustworthiness, educatedness). As such, we could establish whether the three pairs (i.e., “age”–“trustworthiness”; “age”–“educatedness”; and “educatedness”–“trustworthiness”) also related to one another on a perceptual and conceptual level, for each individual participant. As for the group-level analysis, we established the perceptual similarity of pairs of person characteristics by running a Spearman’s correlation on the raw ratings for each participant. We also calculated the conceptual similarity for the relevant three pairs at a participant-level by averaging across the two trials for that pair of characteristics. While previously the person characteristics served as the unit of analysis, in this analysis participants now served as the unit of analysis, with each participant contributing three perceptual and conceptual beliefs similarities. To examine whether perceptual similarity is predicted conceptual similarity ratings, when modelling the data by participants, we ran four linear mixed models for each version of the experiment (typical voices, typical faces, AI-generated voices, and pathological voices) using *lme4* (Bates et al., 2003) in R with conceptual similarity as a fixed effect and participant as a random effect with a random intercept.

## Results

Interrater agreement (as measured by the ICC(2,k)) was good (ICC > 0.7) for most person characteristics and stimulus types, with only Dominance ratings for typical faces and trustworthiness ratings for AI-generated showing lower, moderate agreement (see Table 1). There is no obvious reason within the data to explain why agreement is only moderate for these two characteristics (e.g., no floor or ceiling effects, distributions of responses like other characteristics within this data set). Interrater agreement for conceptual beliefs was excellent (ICC(2,k) = 0.98 across all participants, with ICCs for the participants in each of the four versions of the task exceeding 0.96).

**Table 1:** Interrater agreement (ICC(2,k)) for perceptual ratings for voices and faces

Person characteristic	Typical voices	Typical faces	AI-generated voices	Pathological voices
**Age**	0.95	0.97	0.92	0.93
**Attractiveness**	0.85	0.91	0.88	0.90
**Competence**	0.74	0.81	0.73	0.86
**Dominance**	0.71	0.52	0.82	0.86
**Educatedness**	0.90	0.83	0.76	0.90
**Friendliness**	0.82	0.88	0.73	0.79
**Health**	0.87	0.89	0.86	0.94
**Masculinity**	0.99	0.99	0.99	0.98
**Professionalism**	0.86	0.87	0.81	0.94
**Trustworthiness**	0.79	0.80	0.53	0.83

### Conceptual beliefs and impressions from voices are structured in similar ways

We first asked whether the conceptual belief and perceptual impression spaces derived from typical voices are structured in similar ways. We found strong correlations between the RSMs for conceptual beliefs and impressions from typical voices, Spearman’s rho(45) = 0.63, *p* <.001 (Fig. 1c). Thus, person characteristics that were thought to co-occur in other people on a conceptual level are also more strongly correlated in the context of perceptual impressions inferred from typical voices.

#### The overlap between conceptual beliefs and voice-based impressions is like that of face-based impressions

Next, we sought to directly establish whether our findings for typical voices generalise to and are comparable with similar relationships previously established for typical faces (Stolier, Hehman, Keller et al., 2018b). When correlating RSMs for conceptual beliefs and perceptual impressions from typical faces, we also observe strong positive correlations, with the strength of the relationship being remarkably similar to what we observed for voices, Spearman’s rho(45) = 0.67, *p* <.001 (Fig. 1c). We therefore replicate previous findings for faces (Stolier, Hehman, Keller, et al., 2018b) and further show that the degree of overlap between conceptual beliefs and perceptual impression spaces is similar, independent of stimulus modality. Indeed, we also observe strong correlations between the RSMs for typical voices and faces, Spearman’s rho(45) = 0.74, *p* <.001, underscoring again the generalisable structure of perceptual impression spaces, beyond stimulus set and even modality.

### Beyond “typical” voices: Conceptual beliefs predict impressions for AI-generated and pathological voices

To fully understand how conceptual beliefs and voice-based impressions, it is important to look beyond what is considered “typical.” Prior work already suggests that impressions formed from voices that deviate from a notional (human) norm may differ (e.g., Kühne et al., 2020; Setzen et al., 2023). We therefore extended our analysis to examining impressions from AI-generated and pathological voices. As for typical voices, we observe the same relationships for other types of voices: As such, there we strong correlations between conceptual beliefs and impressions from state-of-the-art AI-generated voices, Spearman’s rho(45) = 0.59, *p* <.001, and pathological voices, Spearman’s rho(45) = 0.66, *p* <.001 (Fig. 1c).

### Conceptual beliefs predict first impressions at an individual level, independent of modality and stimulus type

So far, we have seen converging evidence that for group-level data, there are similarities in the spaces that underpin conceptual beliefs about other people and impressions formed from voices (and faces), independent of which voices are used. Given the group-level data, we can, however, not draw any direct conclusions about how individual form first impressions and how these impressions might relate to their conceptual beliefs. We there next directly examined whether Individual participants’ own perceptual impressions should reflect their conceptual beliefs. Here we find that, as for the previous analyses, individual’s conceptual beliefs about pairs of person characteristics would significantly predict how correlated their perceptual evaluations of these pairs of person characteristics were for typical voices (β = 0.03, *p* =.001), typical faces (β = 0.09, *p* <.001), AI-generated voices (β = 0.06, *p* <.001), and pathological voices (β = 0.03, *p* =.007; Fig. 1d).

An analysis comparing the mean impressions for each person characteristics for the four stimulus types can be found in the Supplementary Materials.

## Discussion

We thus show clear evidence that conceptual beliefs are consistently associated with first impression judgments from voices. This association of conceptual beliefs with first impressions judgments was not only observable at a group level when averaging across individuals’ data but was also reflected when modelling the data by individual participants. We thus show that participants’ own belief structures can be mapped onto how they formed first impressions from voices and beyond. These results have important theoretical implications: While traditional models of voice perception acknowledge the potential for top-down processes interacting with bottom-up processes (Belin et al., 2004; Lavan & McGettigan, 2023), studies more often than not conceptualise voices as signals from which impressions of person characteristics, like dominance or trustworthiness, are derived in a bottom-up, stimulus-driven manner. By showing replicable similarities between representations of conceptual beliefs and perceptual impression, we provide evidence that conceptual beliefs could also shape first impressions. This underscores the need to additionally account for top-down influences in first impression formation, alongside the prevailing focus on bottom-up processes in voice perception in both empirical and theoretical future work.

We also show that the relationship between conceptual beliefs and first impressions is not limited only to “typical” adult voices, as they are often investigated in studies of first impressions. We replicated this relationship when listeners evaluated AI-generated and pathological voices, observing remarkably similar effect sizes. This suggests that our findings are generalisable, such that the relationship between conceptual beliefs and first impressions is similar, independent of the nature of the voices evaluated. This is a surprising finding, considering that 1) average first impression judgments did show differences across voice types, with pathological voices, for example, often being less positively evaluated (see Supplementary Fig. 1, Setzen et al., 2023) and 2) the presence of well-established phenomena, such as AI- or algorithm-aversion (e.g., Cadario et al., 2021; Dang & Liu, 2024; Dietvorst et al., 2015), leading humans to avoid and distrust AI-related products or outputs. The latter might conceivably have affected how listeners evaluate AI-generated voices (e.g., providing globally negatively valenced judgments).

Finally, we replicated and extended prior findings from face perception (Stolier, Hehman, Freeman et al., 2018a, Stolier, Hehman, Keller et al., 2018b), showing that conceptual beliefs are associated with first impressions derived from voices to a similar degree as they do for faces. This consistency of effects across modalities supports the claim that first impressions formation could be partially underpinned by domain-general mechanisms as proposed as part of dynamic interactive theory (Freeman & Ambady, 2011; Freeman et al., 2020).

The current study is not without limitations. First, although our sample of voices was diverse considering some characteristics (e.g., voice type, age), it was limited to talkers with North American accents, reading out sentences. Similarly, all listeners were from the US, with English as their first language. Cross-cultural studies are needed to examine whether the observed relationships generalise across different linguistic and cultural contexts, and how potential differences arise. Furthermore, while RSA provides a powerful tool for comparing representational spaces, it is a correlational analysis approach, such that we cannot establish causality. While dynamic interactive theory (Freeman & Ambady, 2011; Freeman et al., 2020) predicts that conceptual beliefs shape first impressions via top-down influences in the context of our study, where listeners make ad hoc judgments about unfamiliar people, we cannot rule out that, for example, both conceptual beliefs and perceptual impressions reflect the same underlying statistical regularities in the environment. Such regularities could be learned through common experience, without there being any direct influence or causal relationship between the two. Furthermore, experiencing voices could also affect and shape conceptual beliefs, social stereotypes, and learned associations. However, in adult perceivers, as they were tested here, bottom-up processes leading shaping conceptual beliefs and other learned associations are likely to work on a much longer time scale than we sampled here, via short, decontextualised ad hoc judgments. As such, we consider it unlikely that the observed effects reflect (primarily) reflect measurable short-term influences of vocal first impressions shaping our conceptual beliefs. Nonetheless, future work therefore needs to introduce experimental manipulations of conceptual beliefs to formally assess and quantify whether there is any causal impact on impression formation.

If conceptual beliefs can inform perceptual impressions, this raises the questions of why perceivers might use conceptual beliefs to inform first impressions. In the absence of specific and diagnostic information about people we have never met before, perceivers use heuristics and stereotypes as shortcuts to categorise these people (e.g., Fiske & Taylor, 2016). Similarly, overgeneralisation effects or halo effects, where perceivers take one, often valenced, cue (e.g., positive or negative emotional content or personality characteristics) perceived from a voice or face and use it to generalise this cue to other characteristics (Forgas & Laham, 2016; Montepare & Dobish, 2003; Nisbett & Wilson, 1977; Zebrowitz & Montepare, 2008). The conceptual beliefs we have measures can form the building blocks underpinning such overgeneralisation and perhaps even social categorisation effects. These snap judgments often serve the purpose to make sense of people and to ideally help people navigate social interactions. While such heuristics can facilitate these social judgments and first impressions, they are, however, often flawed and can perpetuate biases and discrimination. This raises an important question about how we can potentially mitigate the impacts of these automatic, stereotype-driven impressions, which can be detrimental to perceived and the perceived alike? In workplace settings and beyond, implicit bias and stereotype awareness trainings are popular, which aim to disrupt automatic categorisation by encouraging individuals to reflect on their biases and avoid stereotyped evaluations. While there is some evidence showing that such interventions can be effective, other literature shows mixed evidence (Atewologun et al., 2018, for a review). From the perspective of dynamic interactive theory, these interventions can be seen as a way to impose additional top-down influences on first impressions formation, which may dampen the degree to which conceptual beliefs are used to form first impressions in the first place.

Taken together with previous research, our study, however, underlines that first impressions often provide only a limited insights into who person truly is. While certain aspects of a person’s presentation, such as perceived age or gender expression, can be assessed with some degree of accuracy, first impressions are typically based on “cognitive shortcuts” and can be shaped by cultural norms and stereotypes. As such, first impressions function as rough heuristics that occasionally, and/or partially, align with reality. Often, they can, however, also be entirely inaccurate and potentially harmful to both the perceiver and the perceived. In the end, the most straightforward way of not having to rely on heuristics, is to get to know the person over time, such that first impressions to become a second and eventually lasting impressions.

## Supplementary Information

Below is the link to the electronic supplementary material.

Supplementary file1 (DOCX 2243 KB)

## Data Availability

Data and materials are available online (https://osf.io/nbyr6/?view_only=92ba6df8337347fa93f0b46cb15c23e1). The voice stimuli for typical and pathological voices were not shared as they were extracted from an existing database. This database can be accessed online (https://data.mendeley.com/datasets/9dz247gnyb/1).

## Code availability

The analysis code is available online (https://osf.io/nbyr6/?view_only=92ba6df8337347fa93f0b46cb15c23e1).
