# Supplementary material for: Can conceptual beliefs shape impressions from voices?
Source: Psychon Bull Rev. 2026 Mar 31;33(4):125. doi: 10.3758/s13423-026-02876-w (PMC13038661; doi:10.3758/s13423-026-02876-w)
Supplement: Supplementary file 1 — Supplementary file1 (DOCX 2243 KB) [file 13423_2026_2876_MOESM1_ESM.docx]

# Supplementary Analysis

## Mean ratings for the different person characteristics


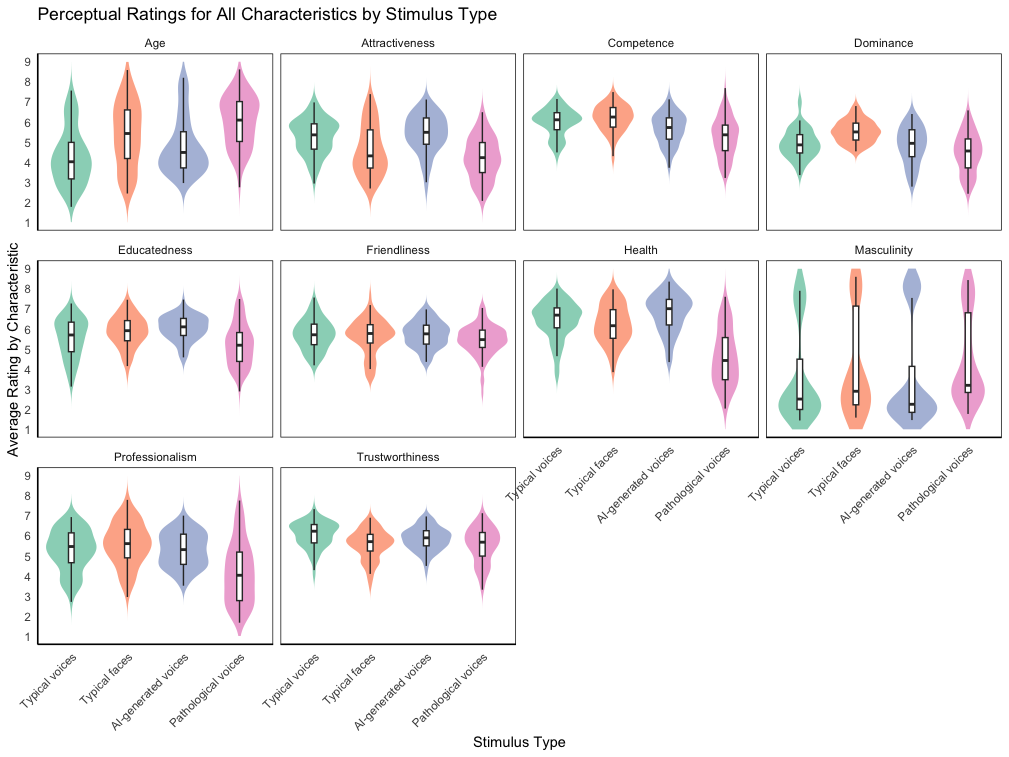


*Supplementary Figure 1. Perceptual Ratings for All Characteristics by Stimulus Type. Violin plots display the distribution of average ratings for the ten characteristics included in this study, Age, Attractiveness, Competence, Dominance, Educatedness, Friendliness, Health, Masculinity, Professionalism, and Trustworthiness, across four stimulus types: Typical voices, Typical faces, AI-generated voices, and Pathological voices. Embedded box plots show the interquartile range and median. Ratings are plotted on a 1–9 scale.*

**Comparing face and typical voices**

When comparing mean ratings per stimulus with typical voices as our "control" condition, independent samples t-tests (p values Bonferroni corrected for 3 comparisons) show that our sample of face stimuli was perceived to be older (t(158) = -4.55, p < .001), less attractive (t(158) = 3.91, p < .001), more dominant (t(158) = -5.79, p < .001), and less trustworthy (t(158) = 4.51, p < .001). There was no significant difference in competence, educatedness, friendliness, health, and professionalism after correcting for 3 comparisons (see Supplementary Figure 1).

**Comparing AI-generated voices and typical voices**

Independent samples t-tests show that, compared to typical voices, our sample of AI-generated voices was perceived to be more competent (t(158) = 2.83, p = .016), more educated (t(158) = -3.72, p < .001), and healthier (t(158) = -2.42, p = .050). There was no significant difference in age, attractiveness, dominance, friendliness, masculinity, or professionalism after correcting for 3 comparisons.

**Comparing pathological and typical voices**

Independent samples t-tests revealed that, compared to typical voices, pathological voices were perceived to be older (t(158) = -8.38, p < .001), less attractive (t(158) = 6.95, p < .001), less competent (t(158) = 5.47, p < .001), less dominant (t(158) = 3.31, p = .004), less healthy (t(158) = 9.77, p < .001), less professional (t(158) = 5.44, p < .001), and less trustworthy (t(158) = 4.33, p < .001). There were no significant differences in perceived educatedness, friendliness, or gender after correcting for 3 comparisons.

## Further details on the representational similarity matrices (RSMs)


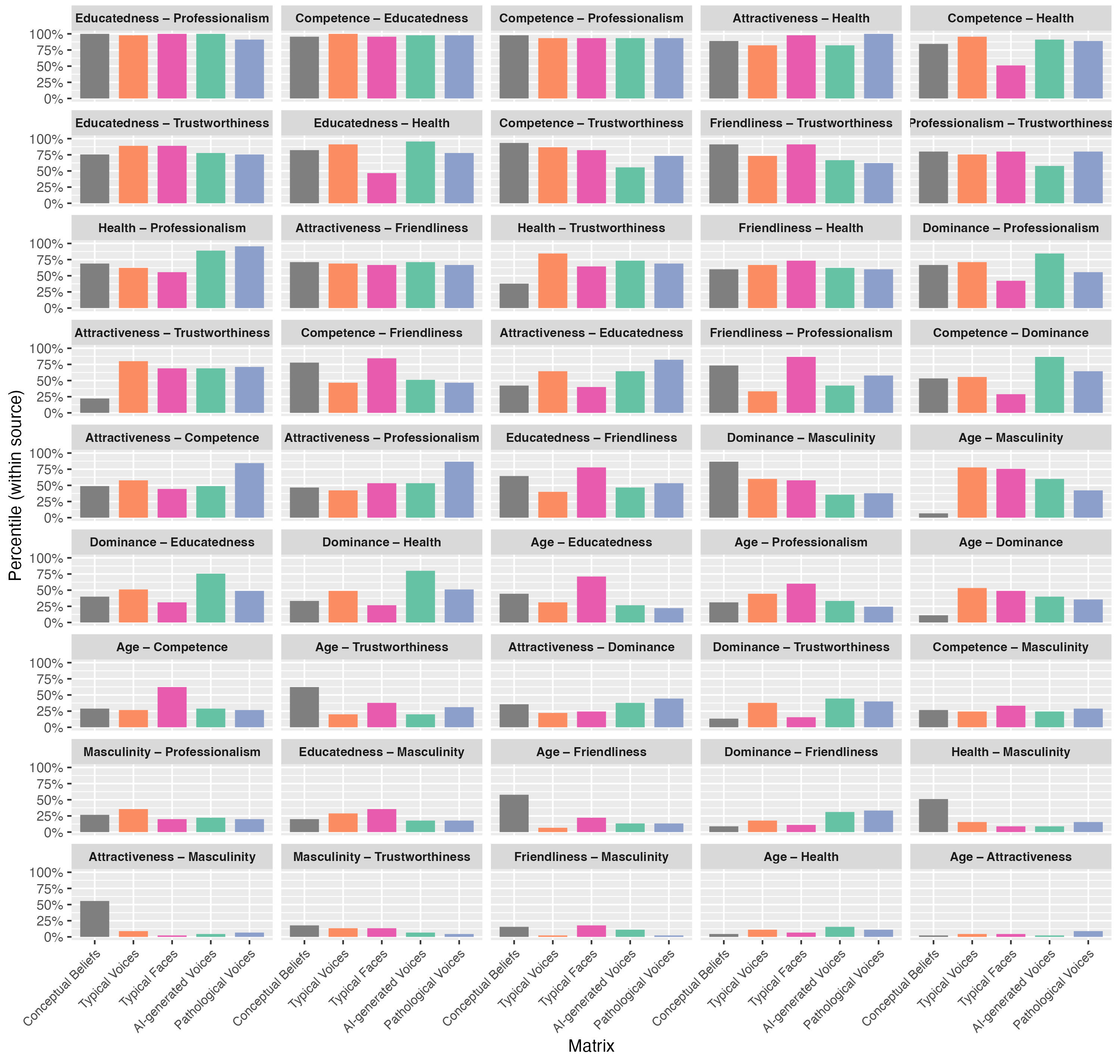


*Supplementary Figure 2: Bars show the percentile rank of each trait-pair associations within its respective representational similarity matrix (RSM), computed over all non-diagonal entries (upper triangle only). This standardization removes differences in scale between matrices (conceptual vs impression ratings), allowing direct comparison of relative strength rather than absolute magnitude. Higher percentiles indicate that a pair of characteristics is among the strongest positive correlations within its respective RSM; lower percentiles indicate weaker and negative correlations.*

Supplementary Figure 2 shows the percentile ranks for the associations for each pair of characteristics within its RSM to allow for easier comparison of association strengths across pairs of characteristics. This plot shows that for many characteristics, there is good agreement in terms of the strength (and direction) of the association between two characteristics. That is, if correlation coefficients are e.g., among the highest within the conceptual beliefs RSM, correlations coefficients will also be high for the impression ratings from all stimulus types (see Educatedness – Professionalism; Age – Attractiveness, etc.). For other pair of characteristics, there is less agreement among the different stimulus types (e.g., Friendliness – Professionalism).
